# The Effects of Zearalenone on the Localization and Expression of Reproductive Hormones in the Ovaries of Weaned Gilts

**DOI:** 10.3390/toxins13090626

**Published:** 2021-09-07

**Authors:** Boyang Wan, Xuejun Yuan, Weiren Yang, Ning Jiao, Yang Li, Faxiao Liu, Mei Liu, Zaibin Yang, Libo Huang, Shuzhen Jiang

**Affiliations:** 1 Department of Animal Sciences and Technology, Shandong Agricultural University, No. 61 Daizong Street, Tai’an 271018, China; sdndwby@163.com (B.W.); wryang@sdau.edu.cn (W.Y.); jiaoning@sdau.edu.cn (N.J.); liyang_cc@yeah.net (Y.L.); 13505388008@163.com (F.L.); liumay@sdau.edu.cn (M.L.); yzb204@163.com (Z.Y.); 2 Department of Life Sciences, Shandong Agricultural University, No. 61 Daizong Street, Tai’an 271018, China; xjyuan@sdau.edu.cn

**Keywords:** zearalenone, gilts, ovary, hormone

## Abstract

This study aims to investigate the effects of zearalenone (ZEA) on the localizations and expressions of follicle stimulating hormone receptor (FSHR), luteinizing hormone receptor (LHR), gonadotropin releasing hormone (GnRH) and gonadotropin releasing hormone receptor (GnRHR) in the ovaries of weaned gilts. Twenty 42-day-old weaned gilts were randomly allocated into two groups, and treated with a control diet and a ZEA-contaminated diet (ZEA 1.04 mg/kg), respectively. After 7-day adjustment, gilts were fed individually for 35 days and euthanized for blood and ovarian samples collection before morning feeding on the 36th day. Serum hormones of E_2_, PRG, FSH, LH and GnRH were determined using radioimmunoassay kits. The ovaries were collected for relative mRNA and protein expression, and immunohistochemical analysis of FSHR, LHR, GnRH and GnRHR. The results revealed that ZEA exposure significantly increased the final vulva area (*p* < 0.05), significantly elevated the serum concentrations of estradiol, follicle stimulating hormone and GnRH (*p* < 0.05), and markedly up-regulated the mRNA and protein expressions of FSHR, LHR, GnRH and GnRHR (*p* < 0.05). Besides, the results of immunohistochemistry showed that the immunoreactive substances of ovarian FSHR, LHR, GnRH and GnRHR in the gilts fed the ZEA-contaminated diet were stronger than the gilts fed the control diet. Our findings indicated that dietary ZEA (1.04 mg/kg) could cause follicular proliferation by interfering with the localization and expression of FSHR, LHR, GnRH and GnRHR, and then affect the follicular development of weaned gilts.

## 1. Introduction

Many toxigenic species of *Fusarium* are the main pathogens of cereal plants, causing head blight in wheat and barley and ear rot in maize. Now, there is a lot of evidence that cereals and animals all over the world are polluted by *Fusarium* mycotoxins, especially ZEA. The trade in these commodities may contribute to the spread of this mycotoxin around the world [1,2]. Zearalenone (ZEA) is a kind of exogenous endocrine disruptor mainly produced by *Fusarium fungi* and widely distributed in maize, wheat, barley and other grain crops [3,4]. ZEA-contaminated feed has posed a widespread threat to animals and human beings due to its high stability during storage and heat treatment [5,6]. ZEA can interfere with hormone metabolism by binding with estrogen receptors, and thereby cause reproductive disorders [7,8]. Pigs are the most sensitive animals to ZEA [9]. During the early ovarian development of piglets, the oocytes developed in the follicles are very vulnerable to the intake of nutrients and environmental estrogens. In turn, the intake of nutrients and environmental estrogens affect the level of hormone metabolism, thus changing the ovarian function [10]. Studies have shown that long-term intake of dietary ZEA can result in swelling of reproductive organs, reproductive disruption, abortion and reduction in litter size [11,12].

Reproductive hormones are an indispensable regulator of reproductive organs development and female reproduction. Gonadotropin releasing hormone (GnRH) is a 10 amino acid peptide secreted by the hypothalamus. It is the initial trigger of hypothalamic-pituitary-gonadal axis (HPG) and plays a crucial role in the occurrence of puberty [13]. It can regulate the synthesis and release of follicle stimulating hormone (FSH) and luteinizing hormone (LH), and then promote follicle maturation and gonadal steroid production [14,15]. Gonadotropin releasing hormone receptor (GnRHR) is a G protein coupled receptor. Previous studies have indicated that GnRH combines with GnRHR on the target cell membrane, and activates the intracellular signaling pathway to complete its biological function [16,17]. Follicle stimulating hormone receptor (FSHR) is an important member of HPG axis, which plays a vital role in granulosa cell (GC) proliferation, apoptosis and differentiation, as well as follicle development and ovulation [18,19]. Luteinizing hormone receptor (LHR) is obtained from growing follicles by the combined action of FSH and estradiol (E_2_). The expression of LHR in the ovarian cycle changes significantly with the alteration of hormone environment, mainly manifested by the changes in FSH and LH levels. It has been reported that FSH, together with other paracrine factors, regulates the development of the primary follicles toward the preantral and antral stages, and a large number of LHR appear under the FSH stimulation [20]. Previous research has studied the localizations or expressions of GnRHR, FSHR and LHR in the ovaries of rats, ewes and rabbits [21,22,23,24,25]. More and more evidence showed that E_2_ and ZEA induced precocious puberty in female rats by increasing the concentrations of serum FSH, LH and GnRH [26,27,28]. The localization of hormone receptors in different cells can better understand the mechanism of early follicular development induced by ZEA. However, the expressions and localizations of ovarian FSHR, LHR, GnRH and GnRHR in the gilts induced by ZEA have not been elucidated.

Therefore, here, this study was conducted to assess the effects of 1.0 mg/kg ZEA on the localizations and expressions of FSHR, LHR, GnRH and GnRHR in the ovaries of weaned gilts, and we hypothesized that ZEA could change the localizations and expressions of FSHR, LHR, GnRH and GnRHR, and then affect the follicular development of weaned gilts.

## 2. Results

### 2.1. Vulva Size

There was no significant difference in the initial vulva area and the final weight between control and the 1.04 mg/kg ZEA treatment (*p* > 0.05, Table 1). The final vulva area, final area/initial area and the final area/final weight of gilts fed the ZEA diet were greater than those of gilts fed the control diet (*p* < 0.05).

### 2.2. Serum Hormones

Serum E_2_, progesterone (PRG), FSH, LH and GnRH levels of gilts are shown in Table 1. Compared with the gilts fed the control diet, gilts fed the 1.04 mg/kg ZEA diet had higher serum levels of E_2_, FSH and GnRH (*p* < 0.05). However, ZEA treatment had no effect on serum PRG and LH levels (*p* > 0.05).

### 2.3. Localizations of FSHR, LHR, GnRH and GnRHR

**Figure 1:**
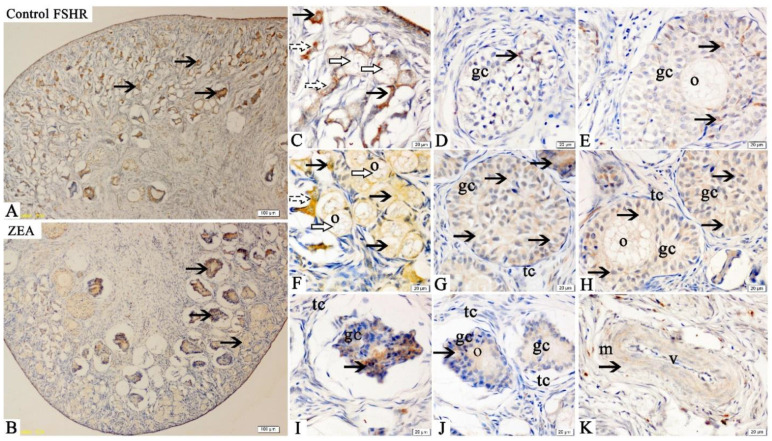
Effects of zearalenone (ZEA) on the follicle stimulating hormone receptor (FSHR) localization in the ovary of weaned gilts. Control (**A**,**C**–**E**) and ZEA (**B**,**F**–**K**) represent the basal diet with an addition of 0 and 1.0 mg/kg ZEA, and with analyzed ZEA concentrations of 0 and 1.04 ± 0.03 mg/kg, respectively. The o, gc, tc, m and v represent oocyte, granulosa cell, theca cell, smooth muscle and vessel, respectively. The hollow arrows indicate the healthy primordial follicle, the dashed arrows indicate the atresia of the primordial follicle, and the black arrows indicate the FSHR localization in the follicle. Scale bars were 100 µm for A and B, and 20 µm for C–K, respectively (*n* = 10).

Immunohistochemical results showed that FSHR immunoreactive substances were mainly localized in the oocytes, GCs and vessel endothelial cells of the ovaries of gilts (Figure 1). Compared with the control group, the positive reactions of FSHR (Figure 1C–J) in oocytes of primordial follicles and granulosa cells of growing follicles in ZEA group were enhanced.

**Figure 2:**
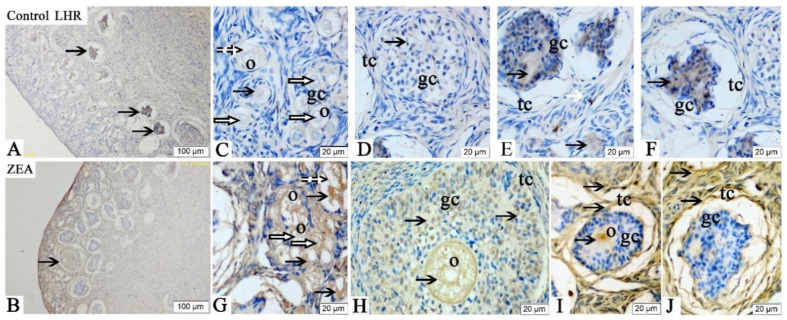
Effects of zearalenone (ZEA) on the luteinizing hormone receptor (LHR) localization in the ovary of weaned gilts. Control (**A**,**C**–**F**) and ZEA (**B**,**G**–**J**) represent the basal diet with an addition of 0 and 1.0 mg/kg ZEA, and with analyzed ZEA concentrations of 0 and 1.04 ± 0.03 mg/kg, respectively. The o, gc and tc represent oocyte, granulosa cell and theca cell, respectively. The hollow arrows indicate the healthy primordial follicle, the dashed arrows indicate the atresia of the primordial follicle and the black arrows indicate the LHR localization in the follicle. Scale bars were 100 µm for (**A**,**B**), and 20 µm for (**C**–**J**), respectively (*n* = 10).

The LHR immunoreactivities were mainly detected in the oocytes, GCs and theca cells of the ovaries in gilts (Figure 2). The overall positive reactions of LHR were weaker than those of FSHR, but the immunoreactive substances of LHR were more obviously observed in the ZEA group compared with the control group (Figure 2C,D,F–H,J). Additionally, the LHR expression was higher in atresia follicles than in healthy growing follicles (Figure 2E,I).

**Figure 3:**
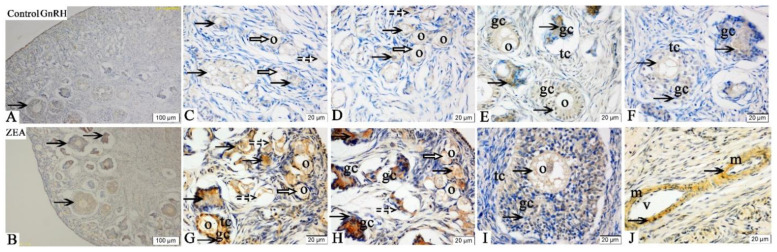
Effects of zearalenone (ZEA) on the gonadotropin releasing hormone (GnRH) localization in the ovary of weaned gilts. Control (**A**,**C**–**F**) and ZEA (**B**,**G**–**J**) represent the basal diet with an addition of 0 and 1.0 mg/kg ZEA, and with analyzed ZEA concentrations of 0 and 1.04 ± 0.03 mg/kg, respectively. The o, gc, tc, m and v represent oocyte, granulosa cell, theca cell, smooth muscle and vessel, respectively. The hollow arrows indicate the healthy primordial follicle, the dashed arrows indicate the atresia of the primordial follicle, and the black arrows indicate the GnRH localization in the follicle. Scale bars were 100 µm for (**A**,**B**), and 20 µm for (**C**–**J**), respectively (*n* = 10).

The immunoreactive substances of GnRH were also mainly detected in the oocytes, GCs and vessel endothelial cells of the ovaries in gilts (Figure 3), which showed that the ovary could secrete the GnRH. Zearalenone consumption increased the GnRH expression in oocytes of primordial follicles and GCs of growing follicles, and the staining intensity in the ovary of the ZEA-treated gilts was significantly stronger than that of the control gilts (Figure 3C,D,G). Meanwhile, the GnRH expression in the atresia follicles was higher than that in the growing follicles (Figure 3E,F,H,I).

**Figure 4:**
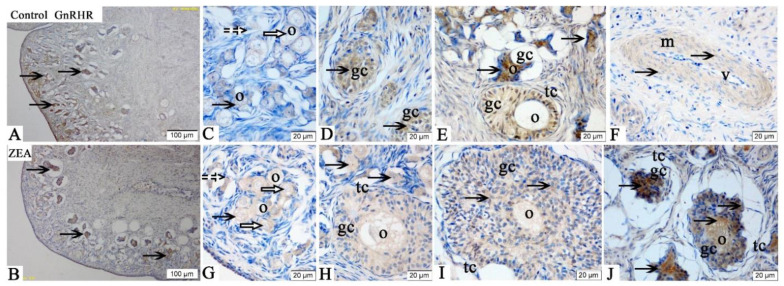
Effects of zearalenone (ZEA) on the gonadotropin releasing hormone receptor (GnRHR) localization in the ovary of weaned gilts. Control (**A**,**C**–**F**) and ZEA (**B**,**G**–**J**) represent the basal diet with an addition of 0 and 1.0 mg/kg ZEA, and with analyzed ZEA concentrations of 0 and 1.04 ± 0.03 mg/kg, respectively. The o, gc, tc, m and v represent oocyte, granulosa cell, theca cell, smooth muscle and vessel, respectively. The hollow arrows indicate the healthy primordial follicle, the dashed arrows indicate the atresia of the primordial follicle, and the black arrows indicate the GnRHR localization in the follicle. Scale bars were 100 µm for (**A**,**B**), and 20 µm for (**C**–**J**), respectively (*n* = 10).

The GnRHR immunoreactivities were mainly detected in the oocytes, GCs and vessel endothelial cells of the ovaries in gilts (Figure 4). Compared with the control group, the positive reactions of GnRHR (Figure 4C–J) in oocytes of primordial follicles and granulosa cells of growing follicles in ZEA group were enhanced.

The results of integrated optic density (IOD) of ovarian FSHR, LHR, GnRH and GnRHR in weaned gilts were consistent with the above results of immunochemical analysis results (Table 2). In general, the IOD of FSHR, LHR, GnRH and GnRHR in the ZEA group were higher than those in the control group (*p* < 0.05).

### 2.4. The mRNA and Protein Expressions

**Figure 5:**
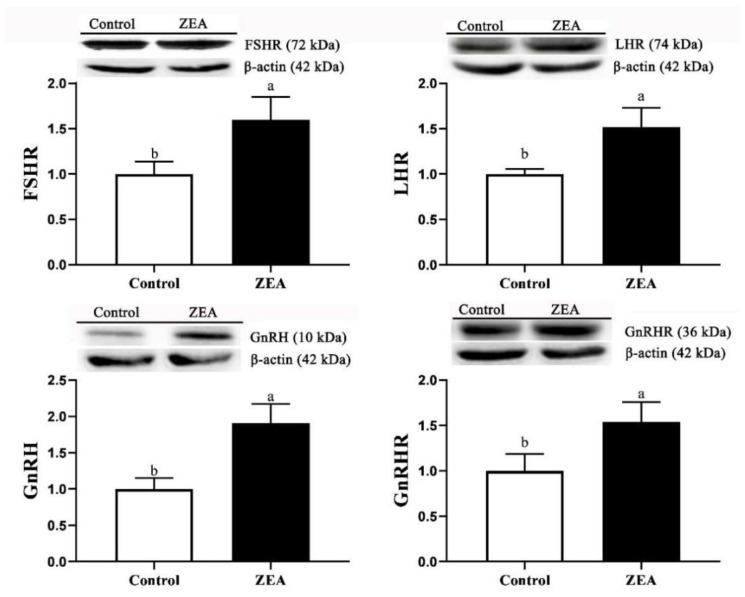
Effects of zearalenone (ZEA) on the protein expressions of follicle stimulating hormone receptor (FSHR), luteinizing hormone receptor (LHR), gonadotropin releasing hormone (GnRH) and gonadotropin releasing hormone receptor (GnRHR) in the ovary of weaned gilts (*n* = 3). Control and ZEA represent the basal diet with an addition of 0 and 1.0 mg/kg ZEA, and with analyzed ZEA concentrations of 0 and 1.04 ± 0.03 mg/kg, respectively. ^a,b^ Means differ significantly (*p* < 0.05).

The mRNA and protein expressions of FSHR, LHR, GnRH and GnRHR in ovaries of weaned piglets are shown in Table 2 and Figure 5. The results were consistent with the result of immunohistochemistry analysis. The relative mRNA and protein expressions of FSHR, LHR, GnRH and GnRHR in the ZEA gilts were significantly higher than those in the control gilts (*p* < 0.05).

## 3. Discussion

Various grains and feeds are pervasively polluted by mycotoxins, which causes serious threats to human and animal wellbeing as well as global commercial trade [8]. It is well-documented that as an exogenous estrogen, ZEA can cause hyperestrogenism in pigs [12]. The clinical symptoms of hyperestrogenism were vulva swelling, prolonged oestrus, anal prolapse and high incidence of stillbirth [29,30,31]. Fu et al. [32] reported that dietary ZEA (1.20 mg/kg) significantly increased vulva width and length of piglets compared to the control group. Similarly, the vulva size of gilts fed with 1.22 mg/kg ZEA increased for a prolonged feeding time [33]. Our previous studies revealed that feeding ZEA-contaminated diets to the gilts (0.96~3.2 mg/kg) could cause vulva swelling and ovarian abnormalities [12,34]. The present results showed that the vulva of weaned gilts fed a ZEA-contaminated diet (1.04 mg/kg) was obviously swollen, suggesting that dietary ZEA may lead to precocious puberty. The most significant change in vulva is mediated by estrogen, which is related to the onset of puberty and the gonadal maturation. In the process of gonadal maturation, follicular development caused the increase in estrogen secretion, which promoted the development of the vulva [35]. It has been reported that endogenous estrogen binds to estrogen receptor subsets and participates in cell proliferation and differentiation through ERK, NF-κB, PI3K/MAPK and other signal transduction pathways [36,37]. However, it is still unclear that whether the mechanisms of ZEA and estrogen leading to vulva swelling is the same, which needs further study.

In the current study, it was successfully observed that dietary ZEA could affect the ovary development by disturbing the reproductive hormones in weaned gilts, which may be highly significant. For early developing animals, mycotoxin poisoning can be determined by examining the changes of serum parameters before clinical symptoms [38]. ZEA and its derivatives can interfere with endocrine, which affects the secretion of steroid hormones [32]. In the present study, we observed that serum E_2_ levels increased by ZEA treated, which was similar to the study of Yang et al. [27]. The main role of E_2_ is to promote the development of female reproductive organs. The increased serum E_2_ concentration was consistent with the appearance of vulva swelling in the present study. The FSH and LH synergistically promotes follicular development and induces the expression of LH receptor on the granulosa cell membrane, which plays a crucial role in the E_2_ secretion by the GCs [20]. A previous study indicated that ZEA (5 mg/kg bw) increased the serum FSH level in female rats [27]. Another investigation in female rats also indicated that ZEA (9 and 13.5 mg/kg BW/d) significantly increased serum FSH level in a dose-dependent relationship [28]. Gao et al. [39] demonstrated the promotional effect of ZEA (20 mg/kg) on FSH secretion in maternal rats. Previous study showed that the positive feedback effect (surge) of E_2_ significantly increased the expression of proto-oncogene Fos in GnRH cells, whereas short-term removal of negative feedback (ovariectomy) has little and the release of GnRH increased in both states [40]. These changed sexual hormones in weaned gilts are considered to be a marker of premature ovarian failure. These results suggest that ZEA interferes with the normal hormone secretion, which is consistent with our present results. However, there was no significant effect in LH concentration when purified ZEA (1~4 mg/kg) was added to the diets of female rats [26]. Similarly, no significant increase was observed in serum concentration of LH in piglets fed diets containing 596.86 μg/kg ZEA [41], which was consistent with our result. Evans et al. [42] found that E_2_ (1.20~7.10 pg/mL) inhibited hypothalamic GnRH secretion in a dose-dependent manner during the time interval between preovulatory luteolysis and gonadotropin surge. It suggests that ZEA interferes with hormone metabolism in a dose-dependent manner. However, it still needs to be further confirmed whether ZEA induced the increased FSH and GnRH by increasing E_2_ level first.

The FSHR, LHR, GnRH and GnRHR were mainly located in the ovarian granular layer and follicular membrane [21]. Similarly, in the present study, the FSHR, LHR, GnRH and GnRHR were found to be mainly located in oocytes and granulosa cells of gilts’ ovaries. The localization and expression of hormone receptors are very important for a better and clearer understanding of the molecular mechanisms and functions of FSHR, LHR, GnRH and GnRHR. In addition, it is worth noting that FSHR, GnRH and GnRHR showed obvious brown immunoreactive substances in the vascular endothelial cells, suggesting that FSHR, GnRH and GnRHR might play roles in blood vessel generation or blood flow regulation in gilts’ ovaries.

FSHR, LHR and GnRHR are all members of the G protein coupled receptor family. Follicle stimulating hormone and LH bind to specific receptors in oocytes, activate intracellular steroidogenic signaling pathway and negatively regulate FSH and LH, thus induced the proliferation and differentiation of ovarian GCs [43,44,45]. The role of GnRHR in ovarian development is mainly to promote the secretion of FSH and LH through the activity of GnRH [46]. Wei et al. [24] reported that Alarelin immunization could stimulate the production of GnRH antibody, inhibit the expression of GnRHR protein, enhance the expressions of FSHR and LHR protein in the ovary, and increase the secretion of FSH, so as to regulate the development of ovary and follicle in ewes. Other, similar research indicated that FSH could promote the development of ovine oocytes, reduce the apoptosis rate, increase the mRNA expressions of FSHR, LHR and GnRHR, and protein expressions of FSHR and GnRHR [24]. ZEA can compete with endogenous estrogen to bind the estrogen receptor, so as to interfere with ovarian gonadal hormone release, which directly affects the expression and function of hormone receptor in female animals, especially animals during reproductive cycle [47]. In our study, the mRNA and protein expressions of FSHR, LHR and GnRHR in the oocytes and GCs of piglets significantly increased by 1.04 mg/kg ZEA treated. In addition, the IOD of FSHR, LHR, GnRH and GnRHR in the ZEA group were higher than those in the control group. To the best of our knowledge, the study is the first to suggest in vivo that dietary ZEA (1.04 mg/kg) can affect the expressions of FSHR, LHR, GnRH and GnRHR, as observed by immunohistochemistry, in the ovary of weaned gilts. Our immunohistochemistry results strongly indicated that hormone receptors play a crucial role in ZEA-induced ovarian dysplasia. Therefore, we speculate that the mRNA and protein expression of GnRHR in the primordial follicles and GCs of gilts are significantly correlated with the protein expression of FSHR and LHR. However, the molecular mechanism needs to be further verified.

In our study, it can be clear that dietary ZEA significantly increased the mRNA and protein expression of GnRH in gilts in a dose-dependent relationship, which was consistent with the previous studies in Kriszet et al. [48] and Yang et al. [27]. GnRH is an important neuropeptide, which can enter pituitary via HPG axis, stimulate the synthesis of LH and FSH, and then regulate the development and function of the gonad [49,50,51]. Therefore, we speculate that ZEA first continuously activates the hypothalamus, upregulates GnRH expression, then induces pituitary to release gonadotropin into serum, and finally leads to vaginal swelling and ovarian weight increase. However, the mechanism of ZEA regulating hypothalamus-pituitary-ovary axis remains to be further confirmed.

## 4. Conclusions

In conclusion, ZEA (1.04 mg/kg) can upregulate the expressions of FSHR, LHR, GnRH and GnRHR in ovaries of weaned gilts, promote the secretion of E_2_, FSH and GnRH, and thereby accelerate vulva swelling and follicular hyperplasia. Therefore, ZEA regulates the development of the vulva and ovary by disordering with the reproductive hormone pathway in gilts. This study laid a foundation for finding sensitive indexes and prevention for targets of reproductive disorders caused by ZEA. However, the mechanisms of ZEA-induced vulva swelling and ovarian development by regulating the hypothalamus-pituitary-ovary axis remains to be further studied.

## 5. Materials and Methods

All agreements used were complied with the Guide for the Care and Use of Laboratory Animals and approved by the Committee on the Ethics (Approval Number: S20180058) of Shandong Agricultural University (Tai’an, China).

### 5.1. Animals, Treatments and Feeding Management

Twenty healthy weaned gilts (Duroc × Landrace × Yorkshire) at 42-day with an average BW of 12.84 ± 0.26 kg were selected and randomly allocated into two treatments, with 10 replicates per treatment. Gilts were housed individually in stainless-steel cages (0.48 m^2^) fitted with plastic slatted floors, feed troughs and nipple drinkers for 35-day test period after 7-day adjustment at the Animal Research Station of Shandong Agricultural University (Tai’an, Shandong, China). During the experimental period, gilts were fed a basal diet (control group) or a ZEA-contaminated diet (the basal diet supplemented with 1.0 mg/kg ZEA). Zearalenone levels used in the present study were based on our previous investigations in Shandong Province of China from 2007 to 2020 and recent literature [12,52,53,54]. The basal diet (Table 3) used in the present study was prepared according to the NRC (2012) [55]. Diets were completed in one batch, sampled and stored in covered containers before feeding. Before the test, the house was cleaned and disinfected. During the first week of the experiment, the room temperature was set to 30 °C, and then maintained between 26 and 28 °C. The relative humidity was approximately 65%.

The nutrients were analyzed according to AOAC (2012) [56]. Mycotoxins were detected by the Qingdao Entry–Exit Inspection and Quarantine Bureau according to the methods of Liu et al. [52], and the minimum detection concentration for ZEA, aflatoxin, fumonisin and deoxynivalenol were 0.01 mg/kg, 1.0 μg/kg, 0.1 mg/kg and 0.05 mg/kg, respectively. The analyzed ZEA contents in the basal diet and ZEA-contaminated diet were <0.01 and 1.04 ± 0.03 mg/kg, respectively, and no other toxins were detected or below the minimum detection concentration.

### 5.2. Vulva Measurement

The length and width of vulva were measured with Vernier caliper every three days to determine the estrogenic effect of ZEA, and the vulva area was approximately calculated as a diamond shape [(vulva length × vulva width)/2] as described by Jiang et al. [57] and Zhou et al. [12].

### 5.3. Serum and Ovary Samples Collection

Gilts were fasted for 12 h on the last day of the feeding trial, and then about 10 mL blood samples were taken from jugular vein into tubes without anticoagulant. The blood was incubated at 37 °C for 2 h and then centrifuged at 3000× *g* for 15 min to obtain the serum, followed by immediately stored in 1.5 mL Eppendorf tubes at −20 °C for the analysis of E_2_, PRG, FSH, LH and GnRH.

Two ovarian samples were isolated from each pig under sterile conditions after euthanasia by electrocution (head only, 110 V, 60 Hz). One of each pair was stored at −80 °C for subsequent analysis of gene and protein expressions of FSHR, LHR, GnRH and GnRHR, and the other was promptly fixed in Bouin’s solution for 24 to 48 h for immunohistochemical analysis. Six ovaries were randomly selected for mRNA expression analysis and three ovaries for protein expression analysis.

### 5.4. Serum Hormone Measurement

Serum levels of E_2_, PRG, FSH, LH and GnRH were determined using radioimmunoassay kits (Nanjing Jiancheng Bioengineering Institute, Nanjing, China) according to method previously described by Jiang et al. [57].

### 5.5. Immunohistochemistry (IHC)

Sections were processed in accordance with the standard IHC protocols. After dewaxing, rehydration and antigen retrieval was performed by microwaving for 20 min at full power in sodium citrate buffer (0.01 mol/L, pH = 6.0). The sections were subsequently treated with 10% hydrogen peroxide (H_2_O_2_) for 1.5 h to deactivate endogenous peroxidase activity and incubated in 10% normal goat serum (ZSGB-BIO, Beijing, China) for 1 h to block nonspecific binding.

The immunohistochemical analysis was performed using a commercial kit (Polink-2 plus^®^ Polymer HRP Detection system for rabbit primary antibody, PV-9001, ZSGB-BIO, Beijing, China) according to the manufacturer’s instructions. Briefly, after washing with phosphate-buffered saline (PBS), the above prepared sections were incubated with anti-FSHR (1:100, bs-0895R, BIOSS, Beijing, China), anti-LHR (1:100, bs-6431R, BIOSS, Beijing, China), anti-GnRH (1:200, 26950-1-AP, Proteintech, Wuhan, China) and anti-GnRHR (1:200, 19950-1-AP, Proteintech, Wuhan China) at 4 °C. The sections were washed with PBS the following day and were subsequently incubated in polymer helper for 1 h at 37 °C followed by Polink-2 plus polymer HRP antirabbit at 37 °C for 1 h. After this incubation, the sections were washed with PBS, followed by immersion in diaminobenzidine tetrachloride (DAB) using a kit (DAB kit, TIANGEN PA110, Beijing, China) for 1~3 min to detect immunostaining. The sections were then dehydrated, sealed in clear resin, mounted and observed microscopically for the localization of immunoreactive substances using a bright field of view.

### 5.6. Integrated Optical Density Measurement

The FSHR, LHR, GnRH and GnRHR labeling was examined by a microscope (Nikon ELIPSE 80i, Tokyo, Japan). Three stained sections of each sample were randomly selected, and three visual fields of each stained section were randomly selected for observation and photography. To estimate the amount of cell staining, the pictures were analyzed using an image analysis software (Image Pro-Plus 6.0, Media Cybernetics, Silver Spring, MD, USA) [58]. Total cross-sectional IOD was acquired, which was used to compare the FSHR, LHR, GnRHR and GnRH staining intensity between control and ZEA treatments. The IOD of each sample is the average of three stained sections.

### 5.7. The mRNA Expression Using Quantitative Real-Time Polymerase Chain Reaction (qRT-PCR)

Total RNA was extracted from the ovaries of gilts using RNAiso Plus (Applied TaKaRa, DaLian, China) according to manufacturer’s instructions and the literature of Song et al. [53] and Zhou et al. [59], and the purity and concentration of the RNA were evaluated by Eppendorf Biophotometer (DS-11, Denovix, Wilmington, DE, USA) at an absorbance ratio of 260/280 nm (a range of 1.8~2.0 indicates a pure RNA sample). The RNA integrity was verified by agarose gel electrophoresis. Total RNA was reverse transcribed to cDNA using a Reverse Transcription System kit (PrimeScript^TM^ RT Master Mix, RR036A, Applied TaKaRa, DaLian, China).

For qRT-PCR, the total volume of the PCR reaction mixture was 20 µL, which contained 10 μL of SYBR Premix Ex Taq-TIi RNaseH Plus (code: RR420A, Lot: AK7502; Applied TaKaRa, DaLian, China), 0.4 μL of both forward and reverse primers, 0.4 μL DyeII, and 2 μL cDNA (<100 ng). The optimized qRT-PCR protocol included an initial denaturation step at 95 °C for 30 s, followed by 43 cycles at 95 °C for 5 s, 60 °C for 34 s, 95 °C for 15 s and 60 °C for 60 s, with a final step at 95 °C for 15 s. The qRT-PCR reactions were conducted in an ABI 7500 Real Time PCR System (Applied Biosystems, Foster City, CA, USA). The relative mRNA was expressed and calculated as equal to 2^−∆∆CT^ [60]. The analysis was repeated three times for each sample. The primer sequences and production lengths are presented in Table 4.

### 5.8. Western Blotting

Ovarian protein was extracted according to the lysate instructions (Beyotime, Shanghai, China), and concentrations were determined using a bicinchoninic acid (BCA) protein assay kit (Beyotime, Shanghai, China) with protein content of each sample being adjusted to 55 µg per sample. The proteins were separated by electrophoresis on polyacrylamide gels and were transferred onto immobilon-p transfer membranes (Solarbio, Beijing, China). The membranes were incubated in 10% skimmed milk for 2 h, washed three times with Tris-buffered saline containing Tween (TBST), and then incubated with primary antibodies: anti-FSHR (1:500, bs-0895R, BIOSS, Beijing, China), anti-LHR (1:500, bs-6431R, BIOSS, Beijing, China), anti-GnRH (1:500, 26950-1-AP, Proteintech, Wuhan, China) and anti-GnRHR (1:500, 19950-1-AP, Proteintech, Wuhan, China), diluted with primary antibody dilution buffer (Beyotime, Shanghai, China), at 4 °C overnight. After washing with TBST, the membranes were incubated with antirabbit IgG (1:1000, Beyotime, Shanghai, China), which were diluted by secondary antibody dilution buffer (Beyotime, Shanghai, China) at 37 °C for 2 h, immersed in a high-sensitivity luminescence reagent (BeyoECL Plus, Beyotime, Shanghai, China), exposed to film using FusionCapt Advance FX7 (Beijing Oriental Science and Technology Development Co. Ltd., Beijing, China), and then quantified using Image software (Image Pro-Plus 6.0, Media Cybernetics, Silver Spring, MD, USA).

### 5.9. Statistical Analysis

Individual piglet was taken as the experimental unit. To determine the difference between control and the ZEA treatment, the data were statistically analyzed using a two-sample pairwise t-test with SAS 9.2 statistical software (SAS Institute Inc., Cary, NC, USA). All data are expressed as the mean ± standard deviation (SD). The difference was considered significant when *p* < 0.05.

## Figures and Tables

**Table 1:** Effects of zearalenone (ZEA) on the vulva size and serum hormones of weaned gilts.

Items	Control	ZEA
Vulva size, cm^2^		
Initial area	1.39 ± 0.14	1.27 ± 0.12
Final area	2.78 ± 0.09 ^b^	4.89 ± 0.18 ^a^
Final weight, kg	29.38 ± 2.60	28.66 ± 2.55
Final area/initial area	2.03 ± 0.25 ^b^	3.89 ± 0.32 ^a^
Final area/final weight, cm^2^/kg	0.10 ± 0.01 ^b^	0.17 ± 0.02 ^a^
Serum hormones		
Estradiol, ng/mL	11.19 ± 0.11 ^b^	14.27 ± 0.10 ^a^
Progesterone, ng/mL	1.23 ± 0.08	1.19 ± 0.06
Follicle stimulating hormone, mIU/mL	2. 87 ± 0.09 ^b^	4.19 ± 0.18 ^a^
Luteinizing hormone, mIU/mL	3.39 ± 0.11	3.62 ± 0.21
Gonadotropin releasing hormone, ng/L	12.29 ± 0.27 ^b^	17.52 ± 0.48 ^a^

Treatments were basal diet supplemented with ZEA at the level of 0 and 1 mg/kg, with analyzed ZEA concentrations of 0 and 1.04 ± 0.03 mg/kg, respectively. Data are mean value ± standard deviation (*n* = 10). ^a,b^ Means differ significantly (*p* < 0.05).

**Table 2:** Effects of zearalenone (ZEA) on the immunoreactive integrated optic density (IOD) and the mRNA expression of ovarian hormones in weaned gilts.

Items	IOD ×10^3^ (*n* = 10)		mRNA Expression (*n* = 6)	
	Control	ZEA	Control	ZEA
Estradiol	5.28 ± 0.36 ^b^	11.33 ± 1.53 ^a^	1.00 ± 0.12 ^b^	3.19 ± 0.12 ^a^
Progesterone	4.32 ± 0.35 ^b^	4.72 ± 0.16 ^a^	1.00 ± 0.09 ^b^	1.53 ± 0.04 ^a^
Follicle stimulating hormone	2.11 ± 0.26 ^b^	4.81 ± 0.34 ^a^	1.00 ± 0.07 ^b^	2.69 ± 0.21 ^a^
Luteinizing hormone	2.89 ± 0.15 ^b^	3.13 ± 0.21 ^a^	1.00 ± 0.13 ^b^	1.96 ± 0.17 ^a^

Treatments were basal diet supplemented with ZEA at the level of 0 and 1 mg/kg, with analyzed ZEA concentrations of 0, 1.04 ± 0.03 mg/kg, respectively. Data are mean value ± standard deviation. ^a,b^ Means differ significantly (*p* < 0.05).

**Table 3:** Ingredients and compositions of the basal diet (as fed basis).

Ingredients	Content (%)	Nutrients	Analyzed Values (%)
Corn	53.00	Metabolizable energy, MJ/kg	13.22
Wheat middling	5.00	Crude protein	19.40
Whey powder	6.50	Calcium	0.84
Soybean oil	2.50	Total phosphorus	0.73
Soybean meal	24.76	Lysine	1.36
Fish meal	5.50	Methionine	0.46
L-Lysine HCl	0.30	Sulfur amino acid	0.79
DL-methionine	0.10	Threonine	0.90
L-threonine	0.04	Tryptophan	0.25
Calcium phosphate	0.80		
Limestone, Pulverized	0.30		
Sodium chloride	0.20		
Premix ^1^	1.00		
Total	100		

^1^ Supplied per kilogram of diet: vitamin A, 3300 IU; vitamin D_3_, 330 IU; vitamin E, 24 IU; vitamin K_3_, 0.75 mg; vitamin B_1_, 1.50 mg; vitamin B_2_, 5.25 mg; vitamin B_6_, 2.25 mg; vitamin B_12_, 0.02625 mg; pantothenic acid, 15.00 mg; niacin, 22.5 mg; biotin, 0.075 mg; folic acid, 0.45 mg; Mn (MnSO_4_·H_2_O), 6.00 mg; Fe (FeSO_4_·H_2_O), 150 mg; Zn (ZnSO_4_·H_2_O), 150 mg; Cu (CuSO_4_·5H_2_O), 9.00 mg; I (KIO_3_), 0.21 mg; Se (Na_2_SeO_3_), 0.45 mg.

**Table 4:** Primers sequences of glycerol triphosphate dehydrogenase (GAPDH), follicle stimulating hormone receptor (FSHR), luteinizing hormone receptor (LHR), gonadotropin releasing hormone (GnRH) and gonadotropin releasing hormone receptor (GnRHR).

Target Genes	Primer Sequence (5′ to 3′)	Product Size	Accession No.
GADPH	F: ATGGTGAAGGTCGGAGTGAA	154	NM_001206359.1
	R: CGTGGGTGGAATCATACTGG		
FSHR	F: ATGTCCTTGCTCCTGGTGTC	213	NM_214386.1
	R: GGTCCCCAAATCCAGAAAAT		
LHR	F: GAAAGCACAGCAAGGAGACC	282	NM_214449.1
	R: ACATGAGGAAACGAGGCACT		
GnRH	F: AGCCAACACTGGTCCTATCGATTG	206	NM_214274.1
	R: GTCTTCTGCCCAGTTTCCTCTTCA		
GnRHR	F: AGCCAACCTGTTGGAGACTCTGAT	101	NM_214273
	R: AGCTGAGGACTTTGCAGAGGAACT		

## Data Availability

Not applicable.
